# Rhizobia–Legume Symbiosis Increases Aluminum Resistance in Alfalfa

**DOI:** 10.3390/plants11101275

**Published:** 2022-05-10

**Authors:** Haifan Shi, Guoli Sun, Lanming Gou, Zhenfei Guo

**Affiliations:** 1 College of Grassland Science, Nanjing Agricultural University, Nanjing 210095, China; shihaifan@njau.edu.cn (H.S.); 20211818@jaas.ac.cn (G.S.); goulanm@njau.edu.cn (L.G.); 2 Jiangsu Coastal Area Institute of Agricultural Sciences, Yancheng 224002, China

**Keywords:** alfalfa, aluminum resistance, nitrogen utilization, nodule, rhizobia

## Abstract

Alfalfa is the most important forage legume with symbiotic nitrogen-fixing nodule in roots, but it is sensitive to aluminum (Al), which limits its plantation in acidic soils. One rhizobia clone of *Sinorhizobium meliloti* with Al tolerance (AT1) was isolated from the nodule in AlCl_3_-treated alfalfa roots. AT1 showed a higher growth rate than the standard rhizobia strain Sm1021 under Al-stressed conditions. Alfalfa growth was improved by inoculation with AT1 under Al-stressed conditions, with increased length and fresh weight in shoots and roots. High nitrogenase activity and pink effective nodules were obtained in AT1-inoculated plant roots under Al stress, with increased total nitrogen compared with the non-inoculated control. The application of exogenous NH_4_^+^-nitrogen increased the Al resistance in alfalfa. It is suggested that rhizobia’s increase of the Al resistance in alfalfa is associated with its improved nitrogen status. Inoculation with Al-tolerant rhizobia is worth testing in an acidic field for improved alfalfa productivity.

## 1. Introduction

Legume–rhizobia symbiosis is the mutual interaction between legumes and rhizobial bacteria that forms a new root organ, i.e., a symbiotic nitrogen-fixing nodule [1]. The atmospheric nitrogen is fixed to form ammonium via nitrogenase in the nodule, while ammonium is assimilated to produce glutamine and glutamate, catalyzed by glutamine synthetase (GS) and glutamine 2-oxoglutarate amino transferase (GOGAT) [2,3,4]. The biological nitrogen fixation not only provides plants with an ecological fertilizer, but also improves the resistance of the host plants to environmental stresses such as drought, dehydration, salt, and alkali stress [5,6,7,8,9].

Aluminum (Al) toxicity is one of the major constraints limiting crop growth and productivity in acidic soils, which occupy approximately 50% of world’s total arable lands [10]. Al^3+^ is extremely toxic to root growth, which in turn causes greatly decreased crop productivity due to impaired water and mineral nutrients absorption from soil [11]. Al toxicity poses an additional challenge to leguminous plants because symbiotic rhizobia are more sensitive to Al than their legume host [12]. Al toxicity reduces rhizobial growth [13,14]. Plant–rhizobia interaction, nodulation and symbiosis nitrogen fixation are delayed or diminished under Al stress [15,16]. Nodulation and nitrogen fixation in soybean under Al toxicity are dependent upon soybean genotypes and Al-tolerant rhizobia improved soybean growth in acidic soils [16,17].

Alfalfa (*Medicago sativa* L.) is the most important forage legume with high nutritional quality and moderate productivity [18,19]. It is sensitive to Al^3+^, which leads to a greatly reduced yield in acidic soils [20,21]. Diversity in Al resistance has been observed among alfalfa cultivars, and higher levels of citrate concentration and exudation are associated with Al resistance in Al-resistant cultivars, as compared with the sensitive cultivars [22]. The toxic effect of Al on alfalfa is alleviated by the foliar application of succinic acid [23]. The overexpression of *neMDH* from alfalfa and a bacterial *CS* leads to promoted organic acid synthesis in roots in transgenic alfalfa plants, and increased Al resistance [21,24]. However, it is unknown whether alfalfa–rhizobia symbiosis affects Al resistance.

The objectives of this study were to isolate Al-tolerant rhizobia, and to examine their influence on Al resistance in alfalfa. A rhizobia strain of *Sinorhizobium meliloti* with Al tolerance was isolated from an Al-tolerant cultivar of alfalfa, and its effect and potential mechanism on increased Al resistance were investigated.

## 2. Results

### 2.1. Isolation and Growth Curve of an Al-Tolerant Rhizobia Strain

**Figure 1:**
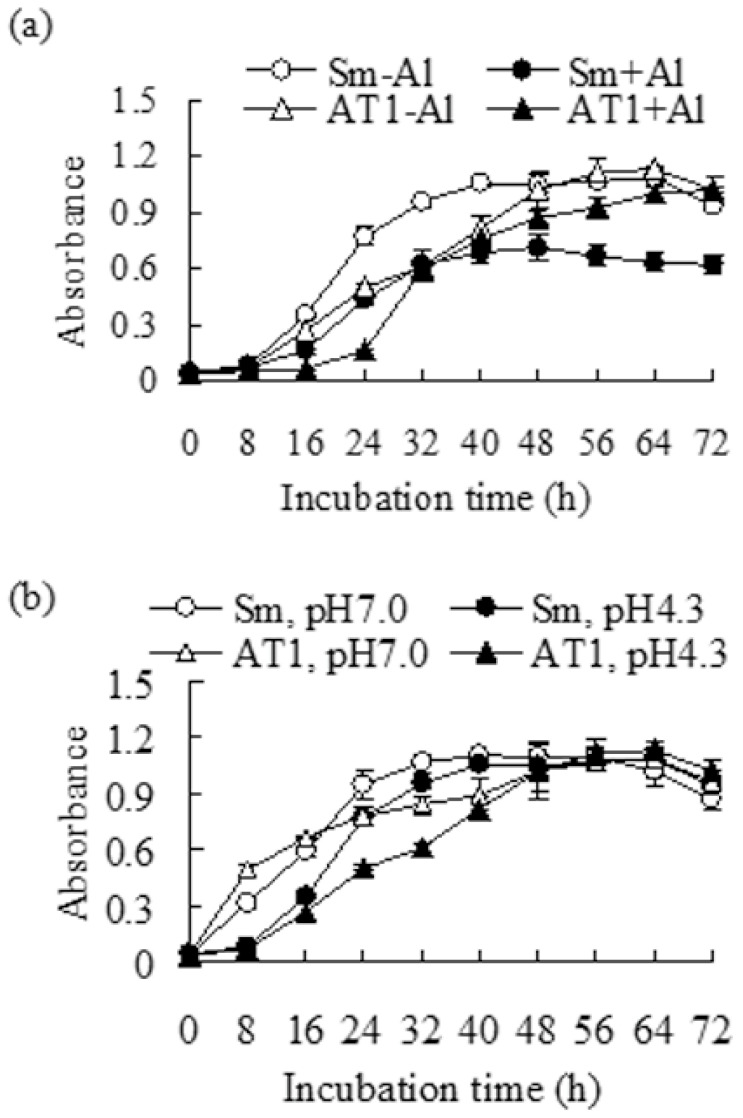
Growth curves of rhizobia strains AT1 and Sm1021. An identical volume of AT1 or Sm1021 was inoculated to the liquid medium (pH 4.3) containing 0.5 mM CaCl_2_ and 1 mM AlCl_3_ (**a**), or the liquid medium adjusting to pH 7.0 or pH 4.3 (**b**). The rhizobia were incubated at 28 °C with shaking. The data presented are means ± SE (*n* = 5).

Nodules were sampled from roots of Al-tolerant alfalfa WL414 growing under Al-stressed conditions in order to isolate Al-tolerant rhizobia [22]. One colony growing on TY agar plates containing 5 mM AlCl_3_ was collected. It was named ‘AT1’ (aluminum tolerance 1). The in vitro growth of AT1 in liquid TY medium containing 0 or 1 mM AlCl_3_ (pH 4.3) was determined to examine Al tolerance. The control rhizobia strain Sm1021 grows faster than AT1 under normal growth conditions (pH 7.0 without Al) within 48 h. The growth of Sm1021 and AT1 was obviously decreased within 24 h under 1 mM AlCl_3_ conditions. Compared the growth under control conditions, Sm1021 remained at lower growth under Al-stressed conditions, while AT1 reached a high growth rate close to the control after 32 h of treatment with Al stress (Figure 1a). In order to examine whether the better growth performance resulted from Al tolerance but not acid tolerance, the growth response of Sm1021 and AT1 to acid was determined under pH 4.3 and pH 7.0 conditions. The growth of both Sm1021 and AT1 was inhibited at pH 4.3 compared with that at pH 7.0 within 24 h. The growth of Sm1021 at pH 4.3 was close to that at pH 7.0 after 32 h, while that of AT1 needed 40 h (Figure 1b), indicating that AT1 was more sensitive to acid than Sm1021. The results suggest that AT1 had a higher Al tolerance than Sm1021.

### 2.2. Alfalfa Growth Was Improved by Rhizobial Inoculation under Low Nitrogen Conditions

**Figure 2:**
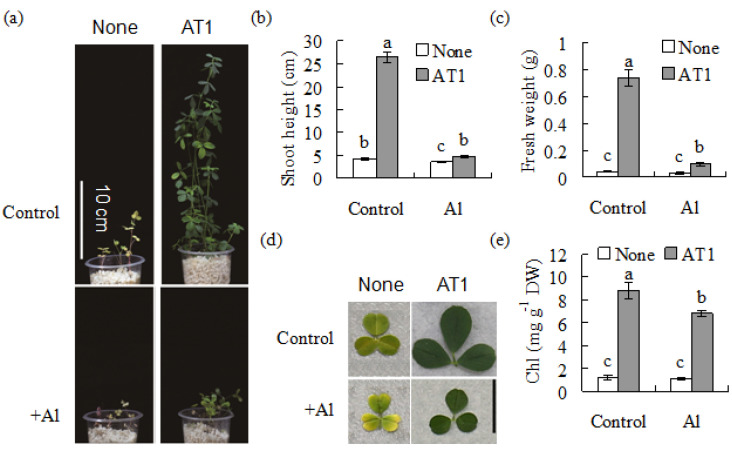
Analysis of the shoot growth in alfalfa, as affected by rhizobia inoculation under Al-stress conditions. An AT1 suspension culture in combination with 1 mM AlCl_3_ or without AlCl_3_ as a control was rhizospherically applied to seedlings of alfalfa 7 d after germination. The low-nitrogen nutrient was maintained at pH 4.3. The photographs of the plants and leaflets were taken after 5 weeks (**a**,**d**), followed by measurements of the plant height (**b**), shoot weight (**c**) and chlorophyll concentration in the leaves (**e**). The data are means ± SE (*n* = 18); the same letter above the column indicates no significant difference at *p* < 0.05.

The plant growth as affected by rhizobia inoculation was examined at pH 4.3 and in low-nitrogen conditions. The alfalfa plants were small in size, with yellow leaves under low nitrogen conditions. The plant growth was improved after inoculation with AT1, with greatly increased shoot height and fresh weight (Figure 2a–c), green leaves (Figure 2d) and increased chlorophyll concentration (Figure 2e). The results indicated that rhizobia inoculation improved the alfalfa growth under low nitrogen conditions. Treatment with 1 mM AlCl_3_ resulted in further reduced growth in alfalfa, while the AT1-inoculated plants showed better growth performance, with increased plant height and fresh weight compared with the non-inoculated plants under Al stress (Figure 2a–c). In addition, the AT1-inoculated plants had a higher chlorophyll concentration than the non-inoculated plants (Figure 2d,e).

**Figure 3:**
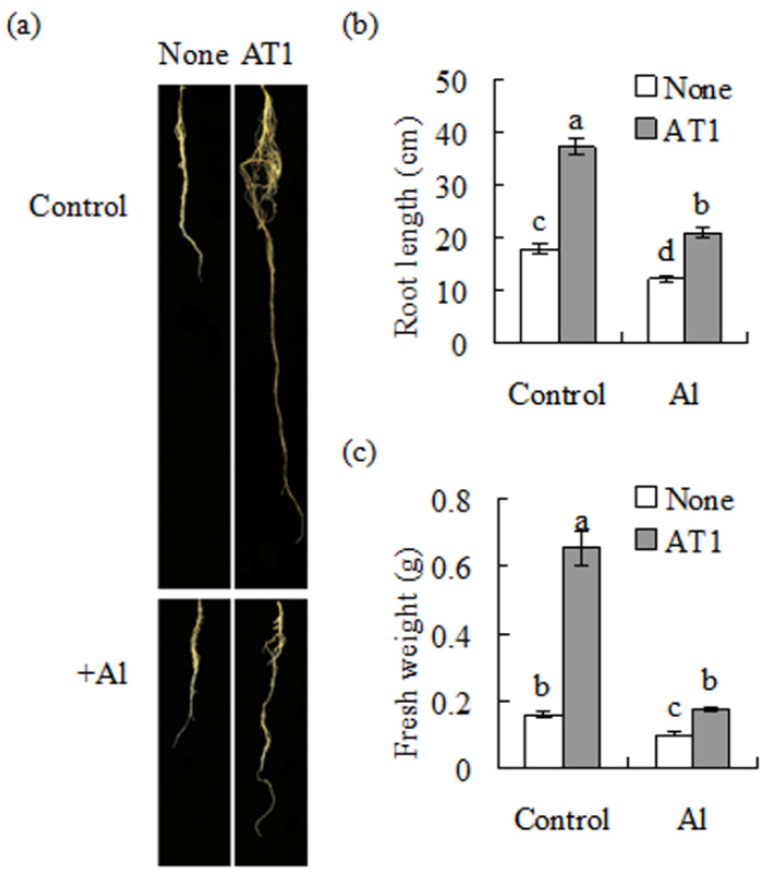
Analysis of the root growth in alfalfa, as affected by rhizobia inoculation under Al stress conditions. The AT1 suspension culture in combination with 1 mM AlCl_3_, or without AlCl_3_ as a control, was rhizospherically applied to seedlings of alfalfa 7 d after germination. The low-nitrogen nutrient was maintained at pH 4.3. Photographs of the roots were taken after 5 weeks (**a**), followed by measurements of the root length (**b**) and root weight (**c**). The data are means ± SE (*n* = 18); the same letter above the column indicates no significant difference at *p* < 0.05.

Root growth was greatly promoted by inoculation with AT1 under low-nitrogen conditions (Figure 3a,b). Treatment with 1 mM AlCl_3_ resulted in the further inhibition of root growth under low-nitrogen condition, while the root length and fresh weight were higher in the inoculated plants than in the non-inoculated plants under Al stress (Figure 3a,c).

### 2.3. Plant–Rhizobia Interaction under Al-Stress Conditions

**Figure 4:**
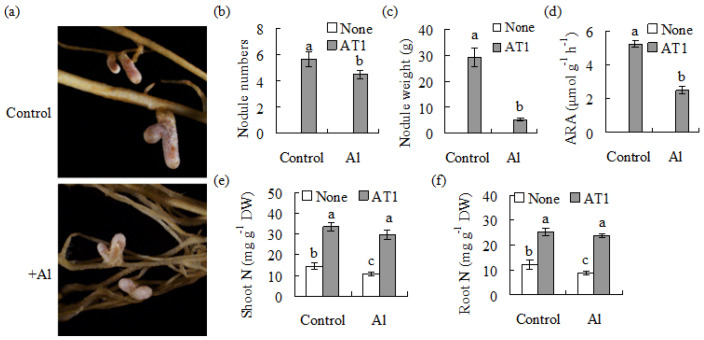
Analysis of the nodulation and plant total nitrogen in alfalfa caused by AT1 under Al stressed conditions. AT1 suspension culture in combination with 1 mM AlCl_3_, or without AlCl_3_ as a control, was rhizospherically applied to seedlings of alfalfa 7 d after germination. The low-nitrogen nutrient was maintained at pH 4.3. Photographs of the nodules were taken after 5 weeks (**a**), followed by measurements of the nodule number (**b**), nodule fresh weight (**c**) and nitrogenase acetylene reduction activity (ARA) (**d**). The shoots (**e**) and roots (**f**) were separately harvested for the measurement of the total nitrogen. The data are means ± SE (*n* = 18). The same letter above the column indicates no significant difference at *p* < 0.05.

In order to assess the effect of AT1 on the symbiosis process under Al-stress, the root nodulation and nitrogen fixation efficiency in alfalfa were analyzed. Elongated and intensely pink nodules were observed on the roots after inoculation with AT1 under low-nitrogen conditions, but their size was decreased under Al-stress conditions (Figure 4a). The nodule numbers, nodule weight and nitrogenase activity were reduced after Al treatment in AT1-inoculated plants (Figure 4b–d). The nitrogen content in plants, as affected by rhizobia inoculation under Al-stress, was determined. The inoculated plants had a higher level of total nitrogen content in their shoots and roots than the non-inoculated plants under the control and Al-stress conditions (Figure 4e,f). The results indicate that rhizobia inoculation’s increase of Al resistance in alfalfa was potentially associated with the improved nitrogen status.

### 2.4. Nitrogen Level Affected Al Resistance

**Figure 5:**
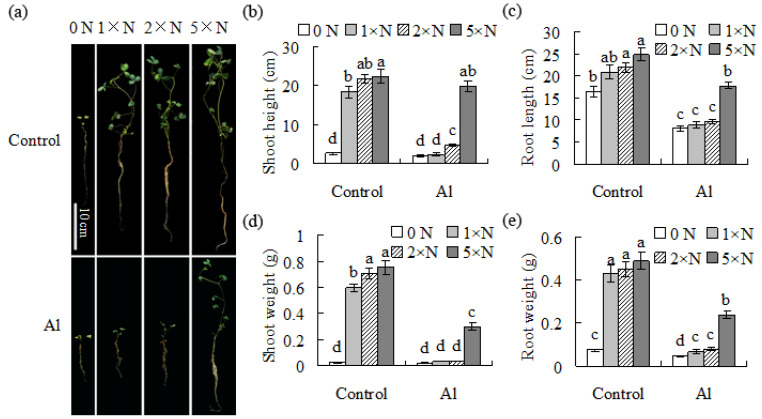
Analysis of the shoot and root growth, as affected by exogenous nitrogen under Al-stressed conditions. One-week-old seedlings of alfalfa were moved to a fresh nutrient solution (pH 4.3) containing 1 mM AlCl_3_ or not, as a control, plus the addition of 0, 0.0267, 0.0534 or 0.1335 g/L NH_4_Cl. After four weeks, photographs were taken of the plants (**a**), followed by measurements of the shoot height (**b**), root length (**c**), shoot weight (**d**) and root weight (**e**). The data are means ± SE (*n* = 30). The same letter above the column indicates no significant difference at *p* < 0.05.

For the demonstration of the proposal, the effect of the application of exogenous nitrogen on Al resistance in alfalfa was measured. Shoots could not grow normally in nitrogen-free nutrient solution, which was improved by the application of 1 N to 5 N ammonia nitrogen in the nutrient solution. The shoot and root length and fresh weight showed no significant difference between the 2 N and 5 N levels, but were slightly greater in 5 N than in 1 N, under control conditions (Figure 5a–e), indicating that the 1 N level is enough for the normal growth of alfalfa. Plant growth was obviously inhibited by treatment with Al under all of the nitrogen levels, while it was better with the increased nitrogen level (Figure 5a–e). Significantly higher levels of shoot and root length and fresh weight were observed in the plants growing in the 5 N nutrient solution than in the others under Al stress (Figure 5a–e). The results suggested that the increased nitrogen supply can enhance Al resistance in alfalfa.

## 3. Discussion

Some rhizobia are recognized to be Al-tolerant bacteria. Rhizobial strains which are tolerant to Al^3+^ are isolated from acidic soils growing beans, cowpea beans and lotus [25,26,27,28]. Some rhizobia strains isolated from amazonian acidic soils can be grown in an acidic liquid medium containing 1 mM Al^3+^ [14]. The *Sinorhizobium meliloti* rhizobia strain RMP5 shows tolerance to 0.1 mM Al^3+^ in a liquid medium [29]. A rhizobia strain of *Sinorhizobium meliloti* AT1 was isolated from nodules of an Al-tolerant alfalfa variety exposed to two weeks of Al stress. AT1 showed good growth performance on a solid medium containing 5 mM Al^3+^, and in a liquid culture containing 1 mM Al^3+^. The Al-tolerance found in rhizobia populations may be partly linked to the decreased amount of negative charge on the cell surface reducing the amount of Al^3+^ bound to the cell [30], neutralizing the effects of Al by forming an insoluble non-toxic complex [31]. A significantly positive correlation between the amount of exopolysaccharide (EPS) and tolerance to Al is observed in some rhizobia [14].

Alfalfa is sensitive to Al toxicity, which greatly limits its plantation in acidic soils [20,22,32]. Al treatment greatly decreased the root and shoot length and fresh weight, while the AT1-inoculated plants showed better performance than the non-inoculated plants, suggesting that rhizobia inoculation improves alfalfa growth and increases Al resistance. This case is similar to soybean, the growth of which in acidic soils is improved by inoculation with Al-tolerant rhizobia [16]. It is suggested that inoculation with Al-tolerant rhizobia like AT1 is worth testing in the field as an effective cultivation measure for improved alfalfa productivity in acidic areas.

Pink nodules were observed in the alfalfa roots after AT1 inoculation under Al-stressed conditions, although the total nodule number and weight were decreased under Al-stress conditions compared with the control. About 50% of the nitrogenase activity of the nodules was also observed in AT1-inoculated plants under Al stress. Nitrogenase plays a key role in biological nitrogen fixation by catalyzing the reduction of N_2_ to ammonia [33]. Nevertheless, our results suggest that AT1 had high nitrogen fixation efficiency under Al-stressed conditions, which led to improved growth in roots and a higher chlorophyll concentration in AT1-inoculated plants compared with the un-inoculated plants. The nitrogen status influenced the Al resistance, which was observed through the application of exogenous nitrogen. The shoot and root length and fresh weight were greatly increased in the 5 N nutrient solution under Al-stressed conditions. Al toxicity on Chinese fir is alleviated by the application of exogenous NH_4_^+^-N but not NO_3_^−^-N with a reduced malondialdehyde level [34]. It is suggested that the increased Al resistance in alfalfa due to rhizobia inoculation is associated with increased nitrogen availability for the plants.

## 4. Materials and Methods

### 4.1. Isolation and Identification of Al-Tolerant Rhizobia from Root Nodules

The alfalfa cultivar ‘WL414′ was grown in a 50-pot tray filled with mixed soil (pearlite: vermiculite: peat =1:1:2, *v*/*v*) for two weeks under natural light in a greenhouse, followed by two weeks of Al treatment by soaking in Hoagland nutrient solution containing 1 mM AlCl_3_ (pH 4.3) for 1 h every day, as previously described [22]. The nodules observed in a few plants were harvested and surface-sterilized in 6.25% NaClO for 30 min, and were washed several times using sterilized water. The bacterial fluid from the crushed nodules was diluted with 1 mL sterile water and then streaked onto TY agar plates (1% tryptone, 0.5% yeast extract, 2 mM CaCl_2_, pH 7.0). After being incubated at 28 °C for 2 d, the single colonies were picked and purified by streaking repeatedly on fresh TY plates containing 5 mM AlCl_3_ (pH 4.3). The single colonies with a stable growth of three generations were activated in TY liquid medium (pH 7.0) in a shaker at 28 °C and 200 rpm/min. The bacterial fluids were amplified with the primers 16 S rDNA (F: 5′-ACTGGCGGACGGGTGAGTAA-3′, R: 5′-CG TATTACCGCGGCTGCTGG-3′) and nif H (F: 5′-CAAGTCSACSACYTCYCARAATAC-3′, R: 5′-AGCATGTCYTCSAGYTCVTCCAT-3′), respectively. The successful amplified sequences were sequenced in order to verify whether the obtained strains were rhizobia strains. The single colony identified as rhizobia which could be grown stably in aluminum-containing medium of rhizobia was collected and stored in a freezer at −70 °C, and was labeled as AT1 (Al tolerance 1).

### 4.2. Analysis of the Al Tolerance of Rhizobia

Rhizobial bacteria Sm1021 or AT1 were incubated in TY medium (3 g/L yeast extract, 5 g/L tryptone, 0.6 g/L CaCl_2_, pH 7.0) with continuously shaking (120 rpm) at 28 °C for 2 to 3 d until OD_600nm_ = 1.0. The bacterial precipitates were collected by centrifugation at 4000 rpm, then re-suspended in sterilized water until OD_600nm_ = 0.2. The rhizobia were added to TY broth (pH 7 or 4.3) containing 1 mM AlCl_3_, or without AlCl_3_ as control, at a ratio of 1:100. The rhizobia growth was recorded by measuring absorbance 600 nm using a spectrophotometer every 8 h over 3 d of incubation at 28 °C, with shaking as above.

### 4.3. Measurement of the Plant Growth and Symbiosis Analysis under Stress Conditions

The sterilized seeds of the Al-resistant alfalfa cultivar ‘WL414′ [22] were germinated on paper towels at 25 °C in a growth chamber and then transferred to a double-bottle growth pot containing sterilized quartz with 150 mL Fåhraeus nutrient solution [35] without nitrogen (pH 6.5) for one week in a greenhouse, under a 16 h light/8 h dark photoperiod, at 25 °C. For inoculation, rhizobial bacteria AT1 were incubated in TY medium (pH 7.0) with continuous shaking (120 rpm) at 28 °C for 2 to 3 d until OD_600nm_ = 1.0. The bacterial precipitates were collected by centrifugation at 4000 rpm, then re-suspended in sterilized water until OD_600nm_ = 0.5. In total, 1 ml of the suspended bacteria was added into quartz. Half of the pots were inoculated with AT1, and the other half were not inoculated, as a control. The plants were then subjected to Al treatment with 1 mM AlCl_3_ in nitrogen-free nutrient solution (pH 4.3), or without AlCl_3_ as a control. After 4 weeks of AlCl_3_ treatment, the roots and shoots were weighed in order to determine their fresh weight. The distance between the plant base and the top of the main stem was determined as the shoot height. The length of the main root was determined as the root length. For the analysis of the symbiosis, the total nodule number and pink nodule number in each plant were recorded to calculate the percentage of effective nodulation.

### 4.4. Measurement of the Chlorophyll Content

Fresh leaves of the third leaflets from the top (0.1 g) were ground in a mortar using a pestle, and were extracted in 10 mL 95% ethanol (*v*/*v*) for 1 h, followed by filtration. The filtrates were diluted using 95% ethanol for the measurement of the absorbance at 663 nm and 645 nm. The chlorophyll concentration was calculated as described previously [36].

### 4.5. Determination of the Nitrogenase Acetylene Reduction Activity and Nitrogen Content

The nitrogenase activity was measured using the acetylene reduction activity assay, as described by Hardy [37]. Nodulated roots attached to plants were transferred into a sealed jar containing 500 µL deionized water; 10% C_2_H_2_ was added to the root atmosphere. After 30 min of incubation, the C_2_H_4_ formed within the gas phase was measured using gas chromatography (HP 6890 Series Gas Chromatograph System, Agilent, Santa Clara, CA, USA).

The total nitrogen content was determined according to the Kjeldahl method described by Watanabe [38]. Shoots and roots were collected and subjected to drying at 105 °C for 15 min and 80 °C for 12 h. After the dry samples were powdered using a grinding machine, 0.2 g of the samples were placed in Buchi tubes and mixed with 5 g of catalyst (potassium sulphate and zinc sulfate heptahydrate mixed in a ratio of 500 g to 50 g) and 100 mL of 98% sulfuric acid per sample. The mixture was digested for 2 h at 420 °C, and the nitrogen was further determined on an automatic Kjeldahl apparatus (Kjeltec 8400, FOSS, Hoganas, Sweden) by automatically distillating and titrating.

### 4.6. Measurement of the Plant Growth in Response to Different Nitrogen Levels

The germinated seeds of WL414 were placed on a plate with holes, floating on the Hoagland nutrient solution [39], at pH 5.8 in a 1.5 L plastic container. After the seedlings were grown for one week at 25 °C, they were subjected to Al treatment with 1 mM AlCl_3_ in modified Fåhraeus nutrient solution containing 0, 0.0267, 0.0534 or 0.1335 g/L ammonium chloride (NH_4_Cl), respectively. The nutrient solution was changed every 3 d. The shoot and root length and fresh weight were measured after four weeks of growth. Each treatment was repeated three times.

### 4.7. Statistical Analysis

All of the measurements were repeated three times. The data were analyzed using one-way ANOVA. Differences among the means of plant lines or treatments were evaluated by Duncan’s test at the 0.05 probability level. All of the statistical analyses were performed using the Statistical Package for the Social Sciences (SPSS 17.0).

## 5. Conclusions

One rhizobia clone showing Al tolerance (AT1) was isolated from the nodule in AlCl_3_-treated alfalfa roots. Inoculation with AT1 improved alfalfa growth under Al-stressed conditions, with improved shoot and root growth. The increased Al resistance in AT1-inoculated alfalfa provided effective nodules with high nitrogenase activity. The application of exogenous NH_4_^+^-N increased the Al resistance in alfalfa. It is suggested that rhizobia’s increase of Al resistance in alfalfa is associated with the improved nitrogen status.

## Data Availability

The data that support the findings of this study are available from the corresponding author upon reasonable request.
